# Genome sequence of *Shigella sonnei* 4303

**DOI:** 10.1186/s13099-018-0274-5

**Published:** 2018-10-24

**Authors:** Laura Deutsch-Nagy, Péter Urbán, Zsuzsanna Tóth, Zoltán Bihari, Béla Kocsis, Csaba Fekete, Ferenc Kilár

**Affiliations:** 1 Institute of Bioanalysis, Medical School, University of Pécs, Szigeti út 12, 7624 Pécs, Hungary; 2 Szentágothai Research Centre, University of Pécs, Ifjúság útja 20, 7624 Pécs, Hungary; 3 Department of General and Environmental Microbiology, Faculty of Sciences, University of Pécs, Ifjúság útja 6, 7624 Pécs, Hungary; 4 Department of Metagenomics, Institute for Biotechnology, Bay Zoltán Nonprofit Ltd. for Applied Research (BAY-BIO), Széchenyi tér 5, 6720 Szeged, Hungary; 5 Department of Medical Microbiology and Immunology, Medical School, University of Pécs, Szigeti út 12, 7624 Pécs, Hungary

**Keywords:** Shigellosis, *Shigella sonnei* 4303, Genome, Lipopolysaccharide biosynthesis

## Abstract

**Background:**

*Shigella* spp. are Gram-negative intracellular pathogenic bacteria belonging to the family *Enterobacteriaceae* and can cause bacterial dysentery, a severe diarrheal disease. The pathophysiological impact of the Gram-negative bacteria is highly related to the composition and structural variability of lipopolysaccharides, the major lipoid components of the outer membrane. Out of the 114 genes involved in the lipopolysaccharide biosynthesis pathway, 47 genes are specific to *Shigella* spp. Changes in the specific genes can lead to loss of the O polysaccharide side chain, resulting in rough (*R*) type bacteria with increased sensitivity to temperature, or hydrophobic antibiotics. The formation of various different lipopolysaccharides or lipooligosaccharides has been observed previously in a mutant line showing altered biological properties, but the genetic background has not been investigated in detail.

**Results:**

The parental strain of the mutant line, *Shigella sonnei* 4303, was subjected to whole genome sequencing to gain a better insight into the structure and biosynthesis of lipopolysaccharides. The sequencing revealed a 4,546,505 bp long genome including chromosomal and plasmid DNA, and the lipopolysaccharide biosynthesis genes were also identified. A comparison of the genome was performed with the phylogenetically closely related, wild type, well characterized, highly virulent strain, *S. sonnei* 53G.

**Conclusion:**

Analysis of the lipopolysaccharide biosynthetic genes helped us to get more insight into the pathogenicity and virulence of the bacteria. The genome revealed high similarities with *S. sonnei* 53G, which can be used as a standard in characterizing the *S. sonnei* 4303’s *R*-type isogenic derivatives.

**Electronic supplementary material:**

The online version of this article (10.1186/s13099-018-0274-5) contains supplementary material, which is available to authorized users.

## Background

Lipopolysaccharides (LPSs) are of importance in bacterial physiology, and also in host-bacteria crosstalk [1]. The pathogenicity of Gram-negative bacteria is influenced by the molecular variability (structures and lengths) of LPSs, e.g., serum sensitivity and biofilm forming ability of Gram-negative bacteria are correlated with the lengths of O sidechains. Previous studies have described that *R*-type bacteria with truncated LPS molecules (so-called lipooligosaccharides—LOSs) are more sensitive to hydrophobic antibiotics [2].

Recent studies suggested that *Shigella sonnei* have become more dominant in developed countries [3]. The history of *S. sonnei* 4303 dates back more than 60 years, when the phenomenon of phase variation in *S. sonnei* was examined [4]. This non-pathogenic strain was formed by plasmid loss from a pathogenic *S. sonnei* phase I. strain, due to the instable nature of the virulence plasmid [5]. Later, intensive studies were carried out on the strain and its *R*-type isogenic derivatives, and the chemical structures and structural variabilities of their lipopolysaccharides and lipooligosaccharides (LOSs) have been described. Several interesting *R* mutants were characterized, including an absolute *R*-type strain (*S. sonnei* 4350) and a strain having truncated LPSs with a d-glycero-d-mannoheptose component incorporated in the structure (*S. sonnei* 4351) [6–12]. The lack of appropriate genome-scale information of the investigated strains, including structurally different LPSs, however, hinders our ability to answer fundamental biosynthetic questions. In order to gain more insight into the mechanism of the LPS/LOS biosynthesis *S. sonnei* 4303 was subjected to whole genome sequencing.

## Methods

The genomic library was made by enzymatic shearing with the Ion Xpress Plus fragment library kit, followed by size selection on a 2% agarose E-Gel SizeSelect Gel (Thermo Fisher Scientific Inc., Waltham, MA USA). The template was prepared with 100 pM of the library on an Ion One Touch 2 system (Thermo Fisher Scientific Inc., Waltham, MA, USA). Samples were loaded on an Ion 316v2 Chip and sequenced on an Ion Torrent PGM sequencer, with the Ion PGM Sequencing 200 Kit v2 (Thermo Fisher Scientific Inc., Waltham, MA, USA) in compliance with the manufacturer’s recommendations. De novo assembly was performed using the SPAdes v3.1 Genome Assembler software [13]. For whole-genome alignment, scaffolds in the draft assemblies were reordered to the *S. sonnei* 53G as reference sequence in Mauve software with default parameters [14]. Sequence annotation was performed using Prokka v. 1.9 [15]. MeDuSa (Multi-Draft based Scaffolder) web server was used for genome scaffolding [16]. The genome sequence of *S. sonnei* 4303 has been deposited in the GenBank under the accession number PRJNA361576.

Phylogenetic analysis was performed with the closest relatives selected by 16S rRNA sequences through NCBI (BLASTn). Phylogenetic analysis was performed by Clustal Omega with default settings [17]. Multiple sequence alignment was completed with *adk*, *fumC*, *gyrB*, *mdh*, *purA* housekeeping genes and rRNA genes. The resulted phylogenetic tree represents 12 *S. sonnei* strains including *S. sonnei* 4303 and an outgroupped strain, *Klebsiella oxytocal* FDAARGOS 355.

Nomenclature of the LPS genes were used according to KEGG database [18].

Detailed methodological strategy is described in Additional file 1.

### Quality assurance

Morphological and biochemical characterization identified the strain as *S. sonnei*. The genomic DNA used for sequencing was isolated from a single colony of the bacteria. The 16S rDNA gene was extracted from the genome using RNAmmer 1.2 server [19]. The identity of the strain was confirmed through BLAST annotation against NCBI microbial 16S database.

## Results and discussion

**Fig. 1:**
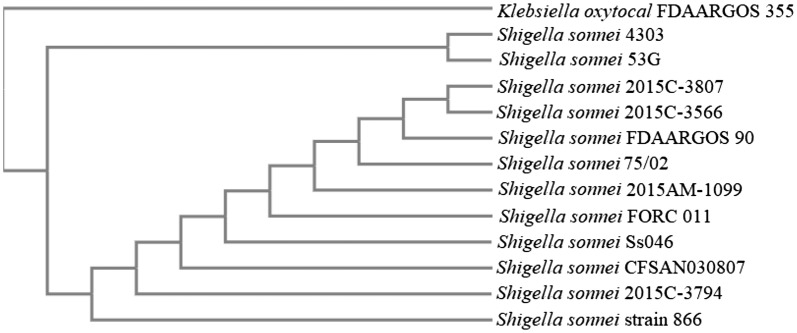
Distance matrix tree showing the phylogenetic relationships of 12 *S. sonnei* strains including *S. sonnei* 4303 and *Klebsella oxytocal* FDAARGOS 355. Phylogenetic analysis was performed by Clustal Omega with 16S rRNA and 5 housekeeping genes (*adk*, *fumC*, *gyrB*, *mdh*, *purA*)

In total 4,262,518 high quality reads were generated and used to create the genome of *S. sonnei* 4303, which yielded a 100-fold coverage. The genome is 4.5 Mbps in size, and contains 4554 predicted genes, 10 rRNA genes, 60 tRNA genes as well as a CRISPR region. In our comparative studies, the genome of a well characterized, highly pathogenic and phylogenetically highly related strain, *S. sonnei* 53G was used as standard (Fig. 1). *S. sonnei* 53G was isolated in Japan [20] and was used in different serological studies [21].

Since the primary aim of this study was to create a solid and strain specific information about the genetic background with regards to LPS modifications, the genes involved in the LPS biosynthetic pathway have been further analyzed in silico. According to the Kyoto Encyclopedia of Genes and Genomes (KEGG) pathways database, 114 genes participate in these complex biological processes. Screening for the presence/absence of these genes in the *S. sonnei* 4303 and in the *S. sonnei* 53G strains revealed 47 genes specific to *S. sonnei*. Comparative DNA analysis on this common subset of *S. sonnei* genes revealed six *s*equence polymorphisms (summarized in Table 1).

**Table 1:** Lipopolysaccharide biosynthesis genes according to Kyoto encyclopedia of genes and genomes in *Shigella sonnei* 4303

Gene name/synonym(s)	Similarity to *Shigella sonnei* 53G (%)
Lipid A	
*lpxB*	100
*lpxA*	100
*lpxD*	100
*lpxL/htrB*	100
*lpxM/msbB*	100
*pagP*	99^a^
*eptA*	100
*lpxC*	100
*lpxH*	100
*arnT*	100
*lpxK*	100
*lpxT/yeiU*	99^b^
*lpxP/ddg*	99^a^
Core region	
*waaA/kdtA*	100
*rfaC/waaC*	100
*rfaF/waaF*	100
*waaQ*	100
*rfaG/waaG*	100
*rfaI*	100
*waaR*	100
*waaV*	99^c^
*waaW*	100
*rfaP/waaP*	100
*rfaY/waaY*	100
*eptB*	100
*eptC*	100
*waaH*	100
O antigen	
*rfaL*	100
*wecA*	100
*wzzB*	100
Unusual sugar	
*kdsD*	100
*kdsA*	99^d^
*kdsC*	100
*kdsB*	100
*gmhA*	100
*gmhC/hldE*	100
*gmhB*	100
*gmhD/rfaD/hldD*	100
*arnA*	99^d^
*arnB*	100
*arnC*	100
*arnD*	100
*arnE*	100
*arnF*	100
*wecB*	100
*wbpA/wecC*	100

^a^Single-nucleotide polymorphism coding nonsense mutation

^b^Two gaps and new stop codon

^c^The gene has 100% similarity to *Shigella sonnei* Ss046’s waaV gene

^d^Single nucleotide polymorphism coding missense mutation

Our former study on the LPS structure of *S. sonnei* 4303 indicated that the lipid A molecules contain only 1 phosphate group at position 1 [6]. Modification of lipid A with an additional phosphate group at position 1, forming a 1-diphosphate species, is mediated by the undecaprenyl phosphotransferase, *LpxT*. The mutation of *lpxT*/*yeiU* encoding gene may explain the monophosphorylated position 1 in *S. sonnei* 4303.

Taken together, the whole-genome sequencing strategy revealed the mutation of the *lpxT*, and the presence of new variants of the *pagP*, *lpxP*, *kdsA* and *arnA* genes. The sequenced genome can be used as a reference for characterizing *R*-type isogenic derivatives of *S. sonnei* 4303, to reveal the genetic background of mutants with the truncated lypopolysaccharides [6], e.g., having a d-glycero-d-mannoheptose in the core part [7, 8].

## Additional file

**Additional file 1.** Complete methodological strategy to the “Genome sequence of *Shigella sonnei* 4303”. Experimental design, Sampling protocol and storage, Nucleic acid isolation, Library preparation and sequencing, Read quality assessment, Comparative genomics.

## Abbreviations

LOS: lipooligosaccharide
LPS: lipopolysaccharide
