# Supplementary material for: Genome sequence of Shigella sonnei 4303
Source: Gut Pathog. 2018 Oct 24;10:47. doi: 10.1186/s13099-018-0274-5 (PMC6201595; doi:10.1186/s13099-018-0274-5)
Supplement: Supplementary file 1 — Additional file 1. Complete methodological strategy to the “Genome sequence of Shigella sonnei 4303”. Experimental design, Sampling protocol and storage, Nucleic acid isolation, Library preparation and sequencing, Read quality assessment, Comparative genomics. [file 13099_2018_274_MOESM1_ESM.docx]

Additional file 1

Complete methodological strategy to the “Genome sequence of *Shigella sonnei* 4303”

- Experimental design

The experimental design followed the description of “Decision Tree for DNA Sequencing on the Ion PGM System” (MAN0007998, Thermo Fisher Scientific Inc., Waltham, MA, USA). After separation of the whole genome with “Qiagen DNeasy Plant Mini Kit”, fragmentation and library preparation was carried out with a manual enzymatic technique, using “Ion Xpress Plus Fragmentation Library Kit” (Publication number 4471989, Revision N). With no barcoding, the library was further modified according to the “Ion OneTouch 200 Template Kit v2 DL” manual (Publication number: MAN0006957, Revision 6.0). Sequencing was performed with “Ion PGM Sequencing 200 Kit” (Publication number 4474246, Revision H).

Qubit 2.0 was used to verify the amount of DNA and Agilent Bioanalyzer 2100 (Agilent, Santa Clara CA, USA) to control the quality of the sample between the steps.

- Sampling protocol and storage

*Shigella sonnei* 4303 was isolated in Pécs, in 1954. Bacterial culture was maintained with passages for short time use. For long-term storage, bacterial cultures were frozen with liquid nitrogen with the addition of 38% glycerol and samples were stored in -80°C.

- Nucleic acid isolation

Nucleic acid was isolated using liquid nitrogen and Qiagen DNeasy Plant Mini Kit (Qiagen, Hilden, Germany). After an overnight cultivation of inoculated LB medium, cells were centrifuged and collected. 4 ml of bacterial cells were transferred to sterile mortar that contained liquid nitrogen. After freezing, bacterial cell walls were disrupted with mechanical strikes.

- Library preparation and sequencing

Library preparation was performed with Ion Xpress Plus Fragmentation Library Kit (Publication number 4471989, Revision N, Thermo Fisher Scientific Inc., Waltham, MA, USA). Library preparation consisted of fragmentation with Ion Shear Plus reagents, ligation of adapters, size selection and amplification. 200 base read libraries were created, from a starting amount of 100 ng gDNA. During enzymatic fragmentation, the optimal reaction time was 15 minutes. Size selection was performed with 2% agarose E-Gel SizeSelect Gel (Thermo Fisher Scientific Inc., Waltham, MA, USA). The template was prepared with 100 pM protocol. Agencourt AMPure XP was used for purification of the fragmented size-selected DNA. The Bioanalyzer 2100 was used to determine the concentration and size distribution of the fragments.

After library preparation, the creation of enriched, template-positive Ion OneTouch 200 Ion Sphere Particles (ISPs) was necessary, which was performed by the Ion OneTouch equipment. The template-positive ISPs containing the clonally amplified DNAs, were sequenced in the Ion Torrent PGM system. For this step we followed the instructions of the manufacturer exactly (Publication number: MAN0006957, Revision 6.0).

- Samples were loaded on an Ion 316v2 Chip. The protocol of the chip loading (Ion PGM Sequencing 200 Kit, Publication number 4474246, Revision H) was slightly modified as it follows; the mixing on the chip and the centrifugation were repeated 3 times (instead of ones, Page 40, step 7-8. in the protocol). Read quality assessment

The quality of the readings was checked using the FastQC quality control tool (Babraham Bioinformatics).

The median of the read lengths was 194 bp, and the total read number was 2,992,965. The alignment of the readings to the reference genome (*Shigella sonnei* 53G) provided a 93% coverage with an average coverage depth of 100.3x.

- Comparative genomics

De novo genome assembly was performed using SPAdes 3.1 Genome Assembler software (St. Petersburg genome assembler) [1] as follows with the command line:

spades --iontorrent --only-assembler -k 21,33,55,77,99,127 -s Shigella_4303.bam -o SPAdesoutput

showing the main commands.

For whole-genome alignment, scaffolds in the draft assemblies were reordered according to the *S. sonnei* 53G as reference sequence in Mauve software [2] applying default parameters.

Prokka 1.9 [3] was used for the genome annotation using the command line:

prokka --genus Shigella --species sonnei --strain Shigella_4303 --usegenus --metagenome --outdir Shigella_4303_Prokka --compliant --prefix Shigella_4303 contigs.fasta

The Mauve and the MIRA4 softwares were used to make the comparative genomic analysis.

The whole genome sequences of *Shigella sonnei 53G*, *Shigella sonnei FDAARGOS_90* and *Shigella sonnei ATCC 29930* strains were used in the MeDuSa web server v 3.1 [4] for genome scaffolding of *Shigella sonnei* 4303.

References

[1] Nurk S, Bankevich A, Antipov D, Gurevich AA, Korobeynikov A, Lapidus A, Prjibelski AD, Pyshkin A, Sirotkin A, Sirotkin Y et al. Assembling single-cell genomes and mini-metagenomes from chimeric MDA products. J Comput Biol. 2013;20(10):714-37

[2] Darling AE, Mau B, Perna NT. ProgressiveMauve: multiple genome alignment with gene gain, loss and rearrangement. PLoS One. 2010;5(6):e11147.

[3] Seemann T. Prokka: rapid prokaryotic genome annotation. Bioinformatics. 2014;30(14):2068-9.

[4] Emanuele B, Beatrice D, Marco G, Sara B, Marie-France S, Pietro L, Pierluigi C, Renato F,Marco F. MeDuSa: a multi-draft based scaffolder. Bioinformatics 2015;31(15):2443-51.
